# Case Report: Disulfiram-induced optic neuropathy

**DOI:** 10.3389/fneur.2026.1793252

**Published:** 2026-04-16

**Authors:** João Mendes, Andrew Fischer, Peter W. Mortensen, Sanjana Jaiswal, Elizabeth Arogundade, Nancy J. Newman, Andrew G. Lee

**Affiliations:** 1 Department of Ophthalmology, Hospital do Espírito Santo de Évora, Évora, Portugal; 2 Department of Ophthalmology, Emory University School of Medicine, Atlanta, GA, United States; 3 Department of Ophthalmology, University of Pittsburgh, Pittsburgh, KS, United States; 4 Department of Ophthalmology, Blanton Eye Institute, Houston Methodist Hospital, Houston, TX, United States; 5 Department of Neurology, Emory University School of Medicine, Atlanta, GA, United States; 6 Department of Neurological Surgery, Emory University School of Medicine, Atlanta, GA, United States; 7 Department of Ophthalmology, Cullen Eye Institute, Baylor College of Medicine, Houston, TX, United States; 8 Department of Ophthalmology, Neurology, and Neurosurgery, Weill Cornell Medicine, New York, NY, United States; 9 Department of Ophthalmology, University of Texas MD Anderson Cancer Center, Houston, TX, United States; 10 Texas A&M College of Medicine, Bryan, TX, United States; 11 Department of Ophthalmology, The University of Iowa Hospitals and Clinics, Iowa, IA, United States

**Keywords:** alcohol use disorder (AUD), central scotoma, disulfiram, drug-induced optic neuropathy, dyschromatopsia, toxic optic neuropathy, vision loss

## Abstract

**Background:**

Toxic optic neuropathy (TON) is characterized by bilateral, symmetric vision loss with damage to the papillomacular bundle, central or cecocentral scotomas, and impaired color vision. While TON has been well-documented with medications such as ethambutol and linezolid, disulfiram-induced optic neuropathy remains rare with limited case reports in the literature.

**Case presentation:**

We report two cases of disulfiram-induced TON in patients with alcohol use disorder. Case 1 involved a 52-year-old man who developed painless, progressive bilateral vision loss after 9 months of disulfiram therapy (250 mg daily). Initial visual acuity was 20/100 OD and 20/70 OS with severe dyschromatopsia (control plate only bilaterally on Ishihara testing) and central scotomas. Case 2 involved a 55-year-old man with 5 years of disulfiram use (250 mg daily) who presented with bilateral central vision loss, visual acuity of 20/50 OD and 20/40 OS, and marked color vision impairment. Both patients underwent extensive workup including neuroimaging, autoimmune panels, infectious serologies, nutritional markers, heavy metal screening, and genetic testing for hereditary optic neuropathies, all of which were unremarkable.

**Results:**

Following disulfiram discontinuation, both patients demonstrated significant visual recovery. In Case 1, visual acuity improved to 20/40 OD and 20/30 OS at 7 months with normalization of visual fields. In Case 2, visual acuity recovered to 20/20 bilaterally at 3 months with complete restoration of color vision (14/14 Ishihara plates). Both cases showed persistent temporal retinal nerve fiber layer thinning on optical coherence tomography despite functional recovery.

**Conclusion:**

These cases highlight disulfiram as an important cause of TON in patients with alcohol use disorder. The differential diagnosis in this population must include nutritional and hereditary optic neuropathies, as mitochondrial dysfunction likely represents a common final pathway. Early recognition and drug cessation can result in substantial visual recovery, although structural changes may persist. Clinicians should maintain awareness of this rare but reversible complication when prescribing disulfiram for alcohol dependence.

## Introduction

Toxic optic neuropathy (TON) is characterized by bilateral, usually symmetric vision loss, damage to the papillomacular bundle, central or cecocentral scotomas, and reduced color vision. The pathophysiology of TON is thought to involve damage to the ganglion cell axons in the papillomacular bundle, resulting in central or cecocentral visual field defects. The differential diagnosis for TON includes nutritional (e.g., vitamin B12 and folate deficiencies), compressive, inflammatory, infectious, demyelinating, and hereditary optic neuropathy (e.g., Leber hereditary optic neuropathy or dominant optic atrophy).

TON is well described with multiple medications (e.g., ethambutol, linezolid). Some toxic optic neuropathies felt to affect mitochondrial function have this pattern of papillomacular bundle involvement, while others such as methanol and amiodarone may feature different patterns of nerve injury. (1) Several reports have described TON due to disulfiram (2–15). Disulfiram, approved for the treatment of alcohol dependence, works by inhibiting aldehyde dehydrogenase. Normally, ethanol is first converted to acetaldehyde by alcohol dehydrogenase. Acetaldehyde is then converted to acetate by aldehyde dehydrogenase. Disulfiram blocks this second step, leading to a buildup of acetaldehyde after alcohol intake. This results in an unpleasant disulfiram-alcohol reaction, with symptoms such as flushing, headache, nausea, vomiting, sweating, vertigo, and lightheadedness. The mechanism of disulfiram-induced optic neuropathy is thought to involve mitochondrial dysfunction, with evidence suggesting that disulfiram causes mitochondrial injury through multiple pathways, including increased reactive oxygen species production, glutathione depletion, and protein insolubility in the mitochondrial matrix. This pathophysiology is similar to other drug-related mitochondrial optic neuropathies affecting the papillomacular bundle.

## Case presentation

### Case 1

A 52-year-old man presented in October 2024 with a 3-month history of painless, progressive bilateral vision loss and dyschromatopsia. His medical history was notable for alcohol dependence with cessation occurring 10 months prior. He denied smoking and reported a healthy diet, and he was taking vitamin supplementation. He had no family history of optic neuropathy or vision loss. His medications included disulfiram 250 mg daily, which he took from December 2023 until September 2024 (total duration 9 months, cumulative dose approximately 67.5 g), discontinuing it 1 month prior to presentation. He denied use of any other medications known to cause optic neuropathy, including ethambutol or linezolid. He had no prior ophthalmological history including cataracts or other conditions that could explain his vision loss. Medication adherence was confirmed to be adequate on patient report and pharmacy records.

On neuro-ophthalmic examination, his visual acuity was 20/100 in the right eye (OD) and 20/70 in the left eye (OS). Ishihara color plates were control plate only in both eyes (OU). Slit-lamp examination was unremarkable OU with no evidence of cataracts. Optical coherence tomography (OCT) revealed a peripapillary retinal nerve fiber layer (RNFL) thickness of 93 μm OU. Automated visual field (24-2) testing showed a mean deviation (MD) of −0.14 dB OD and −0.79 dB OS, with central scotomas OU.

The patient was admitted and started on empiric intravenous corticosteroids. Magnetic resonance imaging (MRI) of the brain and orbits with contrast was normal and showed no evidence of optic nerve or sheath enhancement. Extensive laboratory testing, including autoimmune markers (ACE, lysozyme, anti-aquaporin-4, anti-MOG), inflammatory markers (C-reactive protein, erythrocyte sedimentation rate), infectious serologies (syphilis, tuberculosis, Lyme disease, Bartonella), a hypercoagulability panel (Factor V Leiden, antiphospholipid antibodies, antithrombin III, lupus anticoagulant, functional protein C and S, beta-2 glycoprotein I antibody, anti-cardiolipin antibody, plasminogen, cryoglobulins), serum protein electrophoresis, nutritional markers (vitamins B1, B6, B12, D, folate, methylmalonic acid, homocysteine, calcium, phosphorus, magnesium, copper), and metabolic markers (HbA1c, thyroid panel including TSH, free T4, thyroid-stimulating immunoglobulin, and thyroid peroxidase antibody), were all normal. Heavy metal panel (including arsenic, lead, and mercury) was unremarkable. Phosphatidylethanol testing (Peth; a direct alcohol biomarker to detect prior alcohol use in a window up to 2–4 weeks or more) was negative. Genetic testing for the three most common Leber’s hereditary optic neuropathy mtDNA mutations (Prevention Genetics, MT-ND1/MT-ND4/MT-ND6 Gene Sequencing, Marshfield, WI) as well as a nuclear DNA optic atrophy panel (Prevention Genetics, Optic Atrophy Panel, Marshfield, WI) were negative.

At two-month follow-up from the previous examination, the patient reported subjective improvement in both eyes. Visual acuity was 20/60 OD and 20/30 OS, but Ishihara color vision remained control plate only OU. OCT showed RNFL thickness of 92 μm OD and 94 μm OS. Visual field testing showed MDs of +0.55 dB OD and +0.57 dB OU, with persistent but improved central scotomas OU.

At four-month follow-up (February 2025), the patient continued to improve. Visual acuity was 20/25 OD and 20/30 OS. Visual field testing showed MDs of −0.85 dB OD and −0.01 dB OS, with localized central scotomas OU.

At seven-month follow-up (May 2025), visual acuity was 20/40 OD and 20/30 OS and visual field testing was normal OU.

### Case 2

A 54-year-old man presented with a 4-month history of bilateral simultaneous central vision loss, worsening 1 month prior. His medical history included alcohol use disorder (abstinent for 5 years), hypertension, daily cigar smoking, and 5 years of disulfiram use (250 mg daily, cumulative dose approximately 456 g) along with lisinopril. The patient was adopted, and his family history is unknown. He denied use of other medications known to cause optic neuropathy. He had no prior ophthalmological history including cataracts. Medication adherence was confirmed to be good.

**Figure 1:**
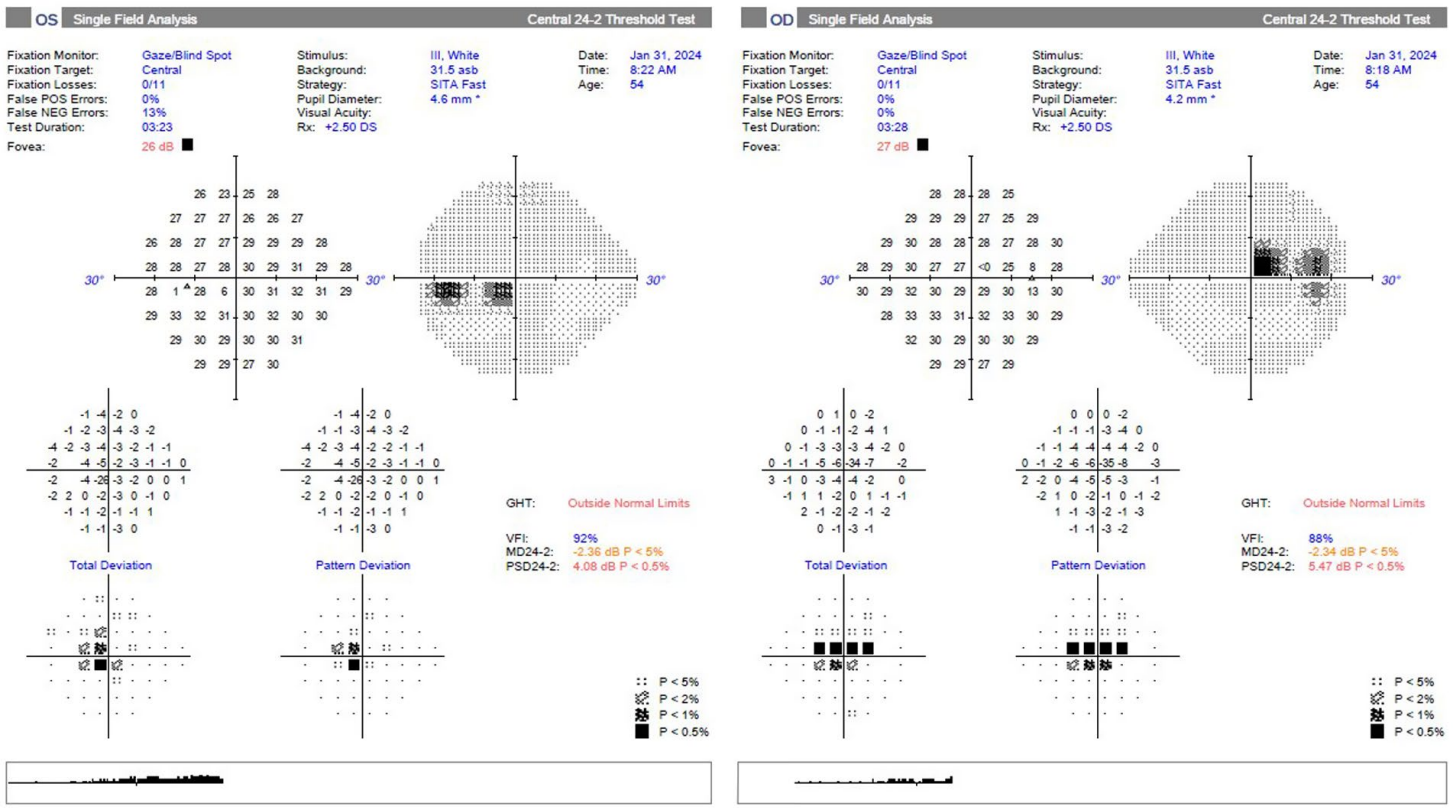
Visual field testing (Humphrey field analyzer 24-2 SITA-fast) at presentation, showing bilateral central scotomas; mean deviations (MD) −2.34 dB OD and −2.36 dB OS.

**Figure 2:**
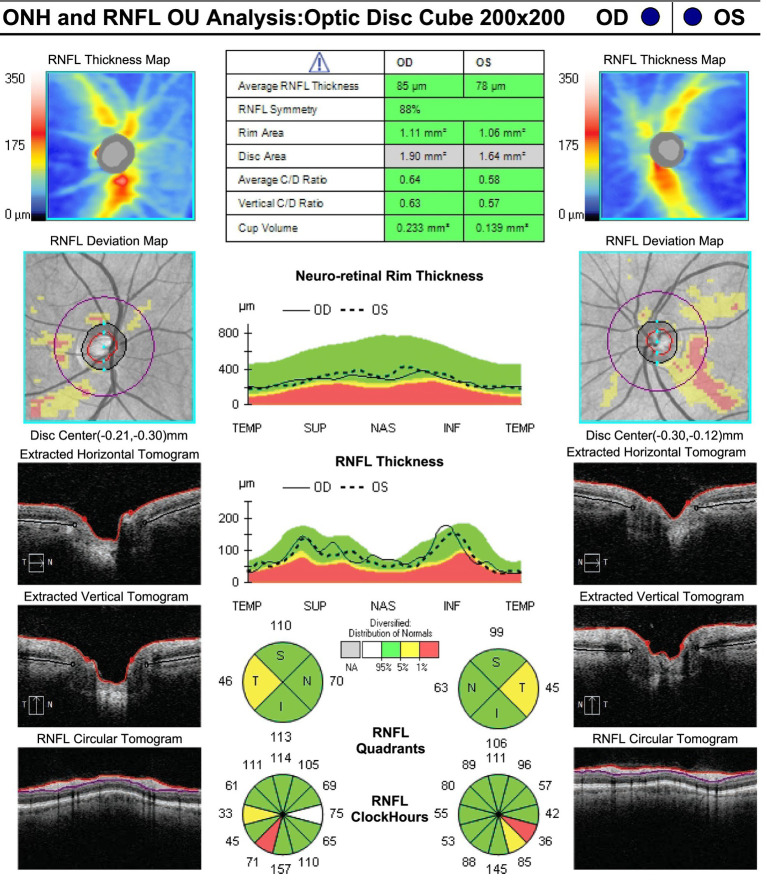
RNFL thickness at presentation: 85 μm OD and 78 μm OS, with bilateral temporal thinning.

**Figure 3:**
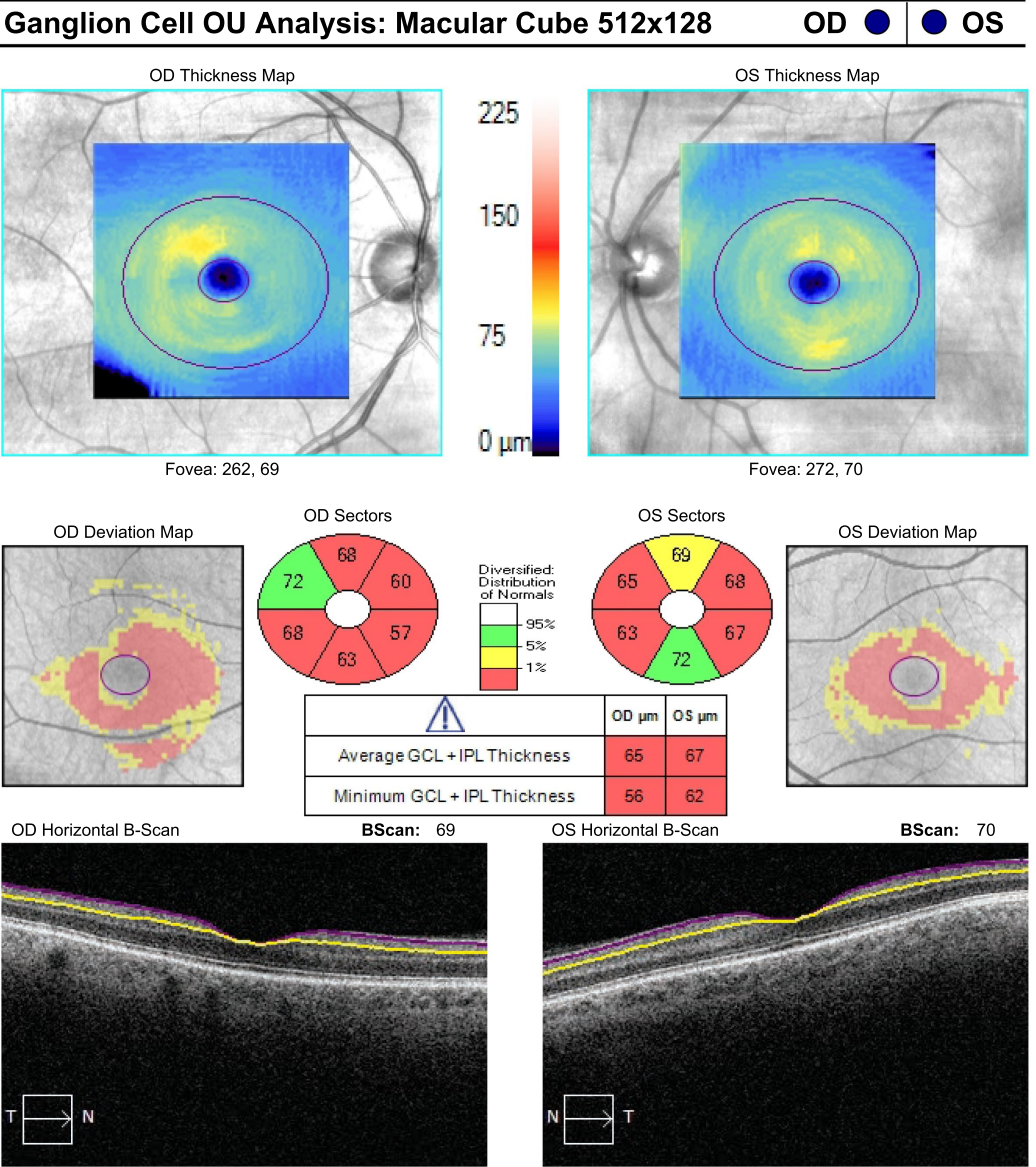
Macular ganglion cell layer at presentation: 65 μm OD and 67 μm OS, showing diffuse thinning.

On neuro-ophthalmic examination, his visual acuity was 20/50 OD and 20/40 OS. No RAPD was present. Ishihara color plates were 7/14 OD and 1/14 OS. Slit-lamp examination revealed no cataracts or other anterior segment pathology. Visual field testing showed bilateral central scotomas, with MDs of −2.34 dB OD and −2.36 dB OS (Figure 1). OCT showed RNFL thickness of 85 μm OD and 78 μm OS, with temporal thinning bilaterally (Figure 2). The macular ganglion cell layer was 65 μm OD and 67 μm OS (diffuse thinning) (Figure 3).

MRI of the brain and orbits with contrast was normal and showed no evidence of optic nerve or sheath enhancement.

Extensive laboratory testing similar to Case 1, including nutritional markers, was negative. Genetic testing for the three most common Leber’s hereditary optic neuropathy mtDNA mutations (Prevention Genetics, MT-ND1/MT-ND4/MT-ND6 Gene Sequencing, Marshfield, WI) as well as a nuclear DNA optic atrophy panel (Prevention Genetics, Optic Atrophy Panel, Marshfield, WI) were negative.

**Figure 4:**
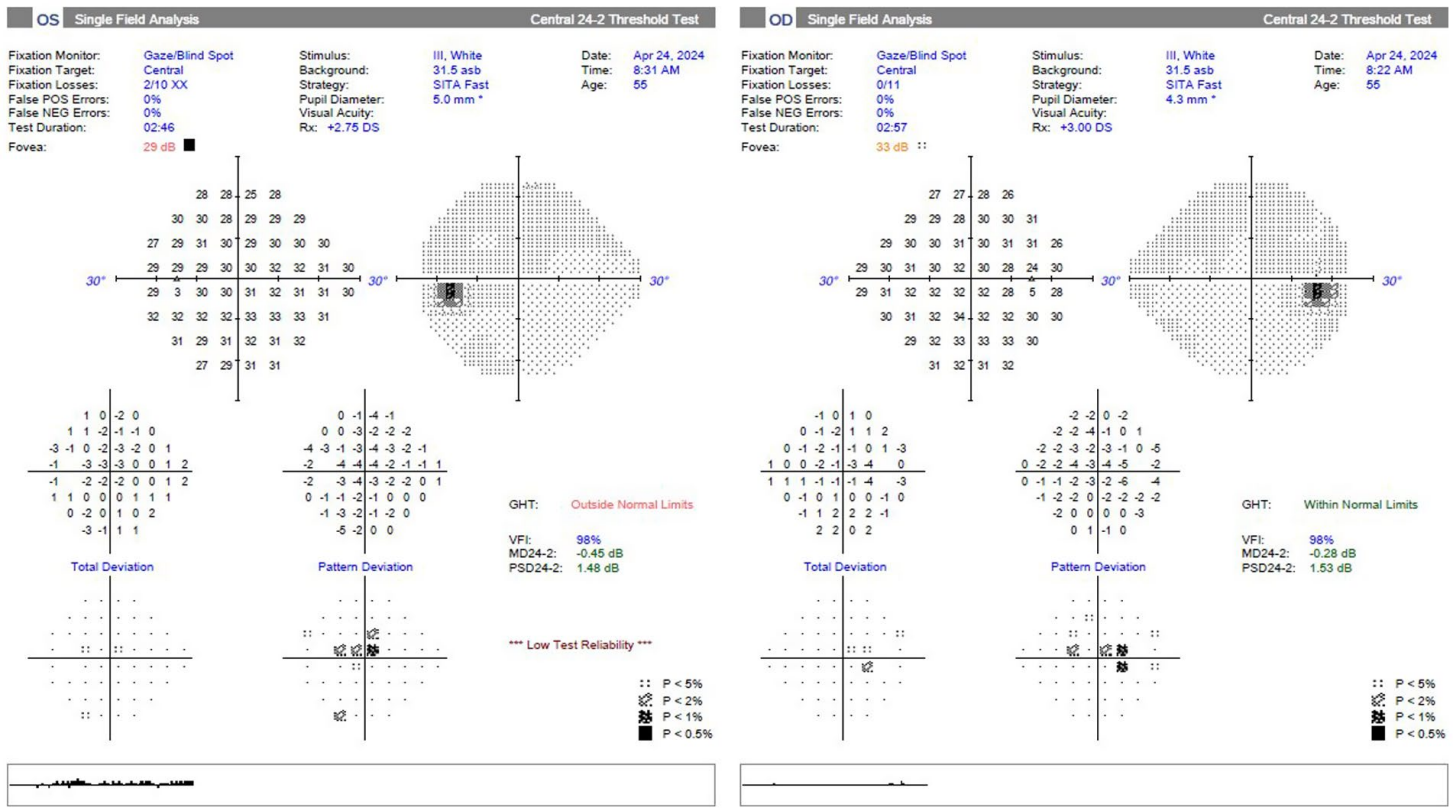
Visual field testing (Humphrey field analyzer 24-2 SITA-fast) at 3-month follow-up, showing improvement of bilateral central scotomas; MDs −0.28 dB OD and −0.45 dB OS.

**Figure 5:**
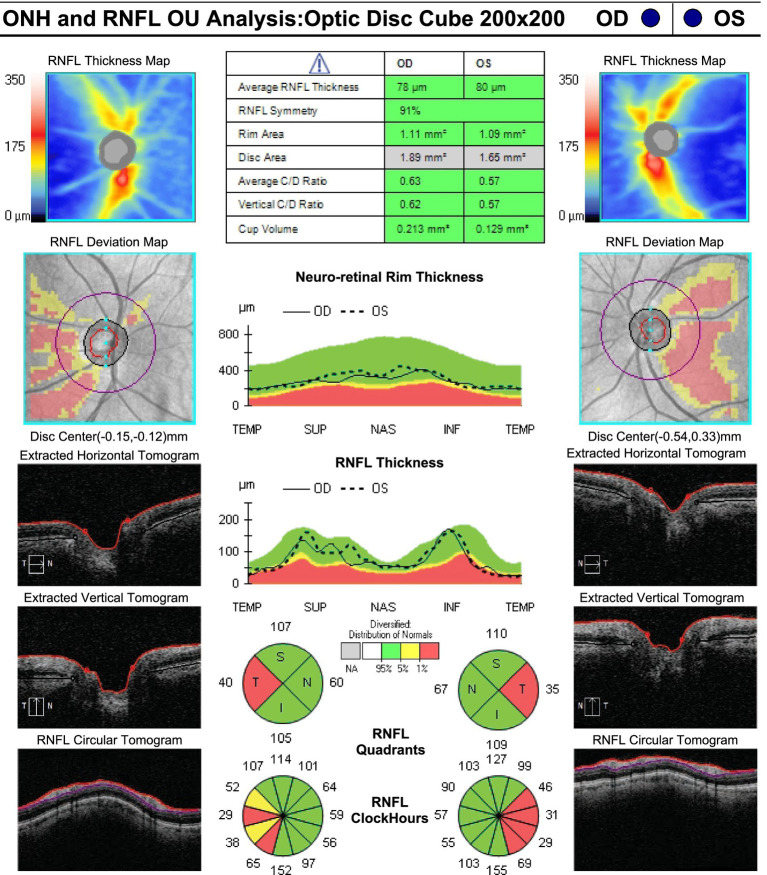
RNFL thickness at 3-month follow-up, showing persistent temporal thinning.

**Figure 6:**
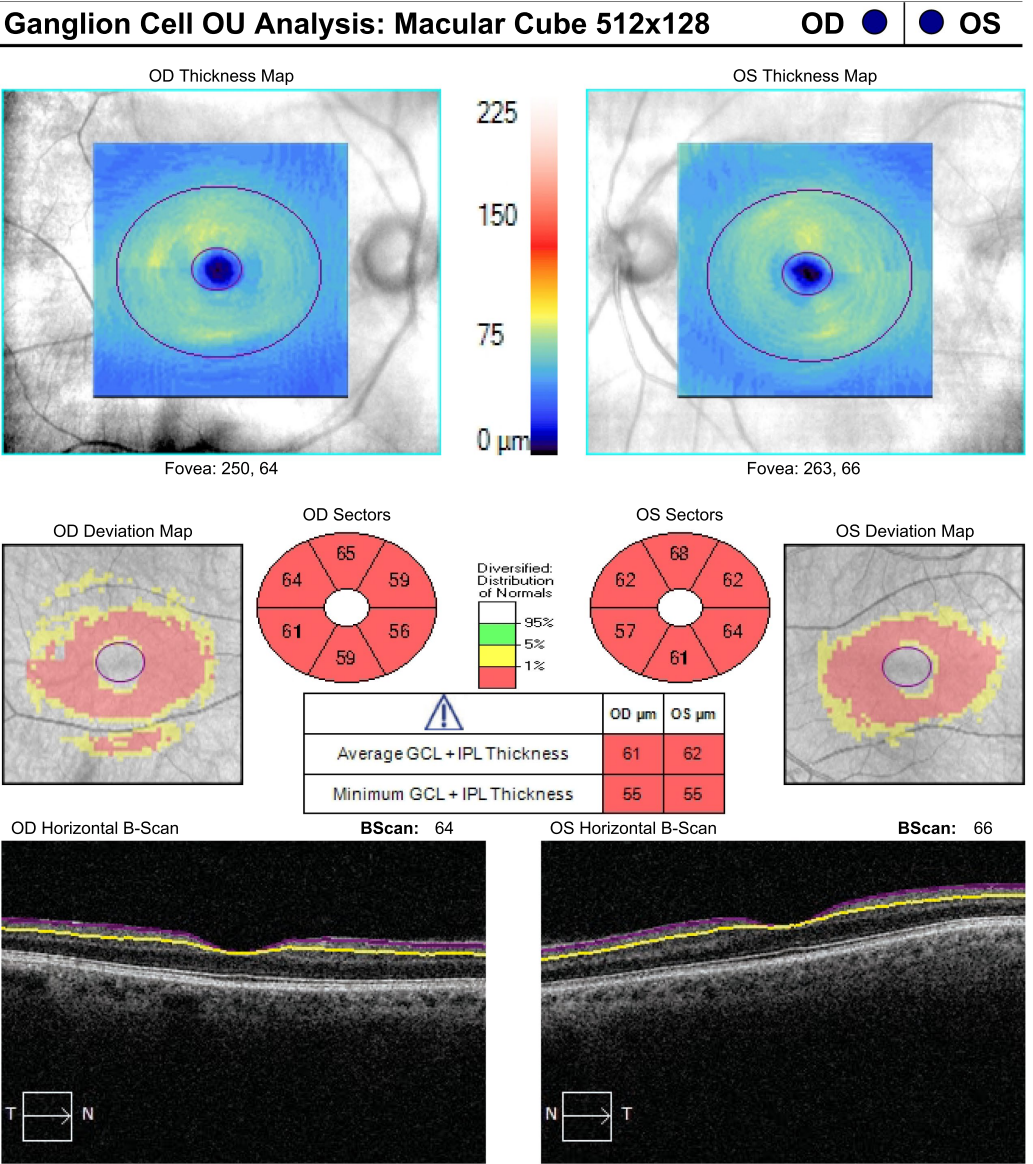
Macular ganglion cell layer at 3-month follow-up, demonstrating diffuse ganglion cell complex thinning in both eyes.

Disulfiram was discontinued, and the patient also ceased cigar smoking at that time. At three-month follow-up, the patient reported visual improvement. His visual acuity was 20/20 OU. Ishihara color plates were 14/14 in both eyes. Visual field testing showed improvement in the bilateral central scotomas, with MDs of −0.28 dB OD and −0.45 dB OS (Figure 4). He had persistent temporal thinning on OCT RNFL as well as diffuse ganglion cell complex thinning in both eyes (Figures 5, 6).

## Discussion

In both cases, visual acuity and visual fields improved following disulfiram cessation. In the patient described in Case 2, both visual acuity and dyschromatopsia recovered rapidly over 3 months, consistent with other cases reported in the literature. In both cases, there was resolution of the central scotomas but persistent temporal thinning on OCT RNFL, indicating permanent structural damage despite functional recovery.

Disulfiram-induced optic neuropathy is a rare condition, with only a limited number of cases reported in the literature (Table 1) (2–15).

**Table 1:** Previously reported cases of disulfiram-associated optic neuropathy.

Publication	Year	Country	Sample size	Initial VA	Final VA
Perdriel and Chevaleraud (2)	1966	France	1	20/50 OU	20/20 (OD) 20/22 (OS)
Saraux and Biais (3)	1970	France	6	20/400 (OU)	20/100 (OD) 20/50 (OS)
				20/200 (OU)	20/100 (OD) 20/40 (OS)
				20/100 (OD) 20/1000 (OS)	20/50 (OD) 20/100 (OS)
				N/A	20/20 (OU)
				20/100 (OU)	No changes
				N/A	N/A
Norton and Walsh (4)	1972	United States	1	20/50 OU	20/30 (OD) 20/20 (OS)
Maugery et al. (5)	1974	France	2	20/400 (OD)	20/20 (OD)
				<20/400 (OS)	20/29 (OS)
Acheson and Howard (6)	1988	United Kingdom	1	20/80 (OD) 20/61 (OS)	20/20 (OU)
Geffray et al. (7)	1995	France	1	20/67 (OU)	20/20 (OU)
Guillaume and Joachim (8)	1998	France	1	20/67 (OD) 20/100 (OS)	20/20 (OU)
Bessero et al. (9)	2006	Switzerland	5	LP (OD)	LP (OD)
				20/67 (OS)	20/22 (OS)
				20/50 (OD) 20/67 (OS)	20/33 (OU)
				20/50 (OD)	20/20 (OD)
				20/400 (OS)	20/67 (OS)
				20/667 (OD) 20/40 (OS)	20/20 (OU)
				20/29 (OD) 20/100 (OS)	20/16 (OU)
Orakzai et al. (10)	2007	Ireland	1	20/50 (OD)	20/25 (OD)
				20/667 (OS)	20/30 (OS)
Hautala et al. (11)	2009	Finland	1	20/25 (OD) 20/33 (OS)	20/25 (OU)
Trélohan and Milea (12)	2011	France	1	20/200 (OD) 20/133 (OS)	20/20 (OD) 20/22 (OS)
Kulkarni et al. (13)	2013	India	1	20/20 (OD) 20/40 (OS)	Improvement OD
Roston and Carey (14)	2024	United States	2	CF (OU)	20/100 (OD) 20/80 (OS)
				20/100 (OD) 20/80 (OS)	20/40 (OD) 20/50 (OS)
Alcalá Torres et al. (15)	2025	Spain	7	20/25 (OD) 20/40 (OS)	20/29 (OD) 20/50 (OS)
				20/200 (OD) 20/25 (OS)	20/200 (OD) 20/22 (OS)
				20/100 (OU)	20/67 (OD) 20/50 (OS)
				20/200 (OD) 20/67 (OS)	20/25 (OD) 20/22 (OS)
				20/200 (OU)	20/67 (OD) 20/100 (OS)
				20/200 (OU)	20/200 (OU)
				20/200 (OD) 20/33 (OS)	20/67 (OD) 20/29 (OS)

CF, counting fingers; LP, light perception; N/A, not available; OD, right eye; OS, left eye; OU, both eyes; VA, visual acuity.

Compared to previously reported cases in the literature (2–15) (Table 2, adapted from Alcalá Torres et al. (15), symptom onset in our series occurred at a mean age of 53 years versus 50.4 years in prior reports. Initial visual acuity averaged 20/60 in our cases compared to 20/78 in the literature, while final visual acuity was similar (20/25 versus 20/30).

**Table 2:** Comparison of our case series with previously reported cases of disulfiram-associated optic neuropathy (15).

Variable	Our series (*n* = 2)	Previously reported cases (*n* = 31)
Sex (% male)	100%	96.8%
Age at symptom onset (years)	53	50.4
Initial visual acuity	20/60	20/78
Final visual acuity	20/25	20/30
Mean treatment duration (months)	34.5	22.7
Mean time to recovery (months)	5	4

Adapted from Alcalá Torres et al. (15).

Time to recovery was comparable, with a median of 5 months in our series versus 4 months in previously reported cases. Treatment duration prior to symptom onset in our cases ranged from 9 to 60 months (mean 34.5 months), compared to a mean of 22.7 months in the literature, with cumulative doses of 67.5 g and 456 g, respectively.

In our series, both patients were on standard maintenance doses of 250 mg daily, but with markedly different durations of exposure: 9 months (cumulative dose 67.5 g) in Case 1 and 60 months (cumulative dose 456 g) in Case 2. Initial visual acuity averaged 20/60 (range 20/100 to 20/40), and final visual acuity averaged 20/25 (range 20/40 to 20/20). Time to recovery was 7 months in Case 1, while Case 2 demonstrated rapid recovery within 3 months.

The relationship between disulfiram dose and duration with the development of optic neuropathy remains incompletely understood. The FDA-approved dosing is typically 500 mg daily for 1-2 weeks initially, followed by maintenance dosing of 125–500 mg daily (average 250 mg daily). Both of our patients were on standard maintenance doses (250 mg daily), but with markedly different durations of exposure (9 months versus 5 years), suggesting individual susceptibility may play a role. Our Case 2 patient had a substantially higher cumulative dose (approximately 456 g) compared to Case 1 (approximately 67.5 g), yet both demonstrated reversibility with drug cessation.

The pathophysiology of disulfiram-induced optic neuropathy likely involves mitochondrial dysfunction, similar to other toxic and nutritional optic neuropathies affecting the papillomacular bundle. Disulfiram has been shown to cause mitochondrial injury through multiple mechanisms including induction of the mitochondrial permeability transition, increased reactive oxygen species production, glutathione depletion, and protein insolubility in the mitochondrial matrix. The papillomacular bundle fibers are particularly susceptible to mitochondrial dysfunction due to their small caliber, lack of myelination, and high energy demands (1, 16, 17).

In patients with alcohol use disorder, the differential diagnosis must include nutritional optic neuropathies (particularly vitamin B12, thiamine, folate, and copper deficiencies), so-called tobacco-alcohol optic neuropathy, and hereditary optic neuropathies such as LHON.

Recent evidence suggests that comprehensive genetic testing, including clinical exome sequencing or gene panel testing for nuclear genes (particularly SPG7, OPA1, WFS1, and ACO2), may be more informative than testing only for the three most common LHON mutations, as genetic susceptibility may predispose certain individuals to toxic optic neuropathy (18).

This approach may identify genetic susceptibility that predisposes individuals to optic neuropathy when exposed to additional mitochondrial stressors such as disulfiram, with important implications for risk counseling and potentially avoiding such exposures (19).

So-called “tobacco-alcohol” optic neuropathy is particularly relevant in this population and represents a confusing overlap of toxic and nutritional factors. Multiple factors may contribute synergistically to visual loss in patients who are alcoholics and smoke, including malnutrition, vitamin deficiencies, and mitochondrial dysfunction.

In Case 2, the patient was a daily cigar smoker, raising the possibility of superimposed toxic effects of smoking contributing to his presentation. However, the temporal relationship with disulfiram use and the dramatic improvement following both disulfiram and smoking cessation suggest disulfiram was the primary causative agent, with smoking potentially acting as a synergistic factor.

## Conclusion

Clinicians should be aware of the possibility of disulfiram TON. In patients with alcohol use disorder, the differential diagnosis includes nutritional and hereditary optic neuropathies, as well as so-called tobacco-alcohol optic neuropathy, especially because the final common pathway of optic nerve compromise is likely via mitochondrial dysfunction (20). Neuroimaging and testing for other causes for optic neuropathy should be performed because there is no definitive test for disulfiram TON. Discontinuation of disulfiram can result in dramatic improvement, but rechallenge is seldom acceptable, even for patients who have benefited from treatment of their alcohol dependency.

Our cases contribute to a better characterization of the clinical picture of disulfiram TON.

The CARE checklist has been completed by the authors for this case report, attached as online Supplementary material.

## Data Availability

The original contributions presented in the study are included in the article/Supplementary material, further inquiries can be directed to the corresponding author.
