# Supplementary material for: Case Report: Disulfiram-induced optic neuropathy
Source: Front Neurol. 2026 Apr 16;17:1793252. doi: 10.3389/fneur.2026.1793252 (PMC13131257; doi:10.3389/fneur.2026.1793252)
Supplement: Supplementary file 1 [file Table_1.docx]

The CARE reporting checklist

For checking that clinical case report articles can be understood and used by everyone

| 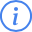 Note |
| --- |
| If you have not used a reporting guideline before, read about [how and why to use them](https:/resources.equator-network.org/about/reporting-guidelines.html) and check whether CARE is the [most applicable reporting guideline](https:/resources.equator-network.org/reporting-guidelines/care/index.html?#applicability) for your work.  Reporting guidelines are most useful when used early in research. When writing a manuscript or application, consider using the [Full Guidance](https:/resources.equator-network.org/reporting-guidelines/care/index.html) where you’ll see explanations and examples for each item.  After writing, demonstrate adherence by completing this checklist:   1. Specify where each item is described (see [Note 1](#sec-specify)). 2. Cite this checklist (See [Note 2](#sec-cite)). 3. Include your completed checklist as a supplement when submitting to a journal so that future readers can use it to find information. |

|  | Item Description | Location (or reason for not reporting) |
| --- | --- | --- |
| **Sections** |  |  |
| [1. Title](https:/resources.equator-network.org/reporting-guidelines/care/items/title.html?utm_source=care&utm_medium=checklist&utm_campaign=CARE_2013_v1_1) | The area of focus and “case report” should appear in the title. | Title |
| [2. Keywords](https:/resources.equator-network.org/reporting-guidelines/care/items/keywords.html?utm_source=care&utm_medium=checklist&utm_campaign=CARE_2013_v1_1) | The key elements of this case in 2–5 words. | Keywords |
| [3. Abstract](https:/resources.equator-network.org/reporting-guidelines/care/items/abstract.html?utm_source=care&utm_medium=checklist&utm_campaign=CARE_2013_v1_1) | 3a – Introduction: What does this case add?  3b – Case presentation:   - The main symptoms of the patient(s). - The main clinical findings. - The main diagnoses and interventions. - The main outcomes.   3c – Conclusion: What are the main “take-away” lessons from this case? | Abstract |
| [4. Introduction](https:/resources.equator-network.org/reporting-guidelines/care/items/introduction.html?utm_source=care&utm_medium=checklist&utm_campaign=CARE_2013_v1_1) | Brief background summary of the case referencing the relevant medical literature. | Introduction |
| [5a. Patient information](https:/resources.equator-network.org/reporting-guidelines/care/items/patient-information.html?utm_source=care&utm_medium=checklist&utm_campaign=CARE_2013_v1_1) | 5a – Demographic information of the patient (age, gender, ethnicity, occupation).  5b – Main symptoms of the patient (chief complaint).  5c – Medical, family, and psychosocial history—including lifestyle and genetic information whenever possible, details about relevant comorbidities, and past interv… | Case discussion |
| [6. Clinical findings](https:/resources.equator-network.org/reporting-guidelines/care/items/clinical-findings.html?utm_source=care&utm_medium=checklist&utm_campaign=CARE_2013_v1_1) | Describe the relevant physical examination (PE) findings. | Case discussion |
| [7. Timeline](https:/resources.equator-network.org/reporting-guidelines/care/items/timeline.html?utm_source=care&utm_medium=checklist&utm_campaign=CARE_2013_v1_1) | Depict important date and times in this case (table or figure). | Case discussion |
| [8. Diagnostic assessment](https:/resources.equator-network.org/reporting-guidelines/care/items/diagnostic-assessment-and-diagnosis.html?utm_source=care&utm_medium=checklist&utm_campaign=CARE_2013_v1_1) | 8a – Diagnostic methods (e.g., physical examination, laboratory testing, imaging, questionnaires)  8b – Diagnostic challenges (e.g., financial, language, or cultural)  8c – Diagnostic reasoning including other diagnoses considered  8d – Prognostic characteristics (e.g., staging) where applicable. | Case discussion |
| [9. Therapeutic Intervention](https:/resources.equator-network.org/reporting-guidelines/care/items/therapeutic-interventions.html?utm_source=care&utm_medium=checklist&utm_campaign=CARE_2013_v1_1) | 9a – Types of intervention (e.g., pharmacologic, surgical, preventive, self-care)  9b – Administration (e.g., dosage, strength, duration)  9c – Changes in intervention (with rationale). | Case discussion |
| [10. Follow up and outcomes](https:/resources.equator-network.org/reporting-guidelines/care/items/follow-up-and-outcomes.html?utm_source=care&utm_medium=checklist&utm_campaign=CARE_2013_v1_1) | 10a – Clinician and patient-assessed outcomes  10b – Important follow-up test results (positive and negative)  10c – Intervention adherence and tolerability (and how this was assessed)  10d – Adverse and unanticipated events. | Case discussion |
| [11. Discussion](https:/resources.equator-network.org/reporting-guidelines/care/items/discussion.html?utm_source=care&utm_medium=checklist&utm_campaign=CARE_2013_v1_1) | Discussion (including conclusion):  11a – Strengths and limitations of the management of this case  11b – Relevant medical literature  11c – Rationale for conclusions (including assessment of cause and effect)  11d – Main “take-away” lessons of this case report. | Case discussion |
| [12. Patient perspective](https:/resources.equator-network.org/reporting-guidelines/care/items/patient-perspective.html?utm_source=care&utm_medium=checklist&utm_campaign=CARE_2013_v1_1) | When appropriate patients should share their perspectives on the treatments they received. | Case discussion |
| [13. Informed consent](https:/resources.equator-network.org/reporting-guidelines/care/items/informed-consent.html?utm_source=care&utm_medium=checklist&utm_campaign=CARE_2013_v1_1) | Did the patient give informed consent? Please provide if requested. | Informed consent |

## 1 How to specify where content is

Tell the reader where they can find information. E.g.,

- Results; paragraph 2
- Methods, Participants; paragraphs 1 & 2.
- Table 3
- Supplement B, para. 4

If you have chosen not to describe an item, explain why. You can do this in the checklist, or as a note below it.

You can describe items in the article body, or in tables, figures, or supplementary materials, and should prioritize items you feel are most important to your intended audience. The order of items in your manuscript does not need to match the order of items in this checklist. You can decide how best to structure your work.

## 2 How to cite

Describe how you used CARE at the end of your Methods section, referencing the resources you used e.g.,

‘We used the CARE reporting guideline(1) to draft this manuscript, and the CARE reporting checklist(2) when editing, included in supplement A’

If you use a reporting checklist, remember to include it as a supplement when publishing so that readers can easily find information and see how you have interpreted the guidance.

1. Gagnier JJ, Kienle G, Altman DG, Moher D, Sox H, Riley D, et al. The CARE guidelines: Consensus-based clinical case reporting guideline development. Case Reports [Internet]. 2013 Oct;2013:bcr2013201554. Available from: <https://casereports.bmj.com/content/2013/bcr-2013-201554>

2. Gagnier JJ, Kienle G, Altman DG, Moher D, Sox H, Riley D, et al. The CARE reporting checklist. In: Harwood J, Albury C, Beyer J de, Schlüssel M, Collins G, editors. The EQUATOR network reporting guideline platform [Internet]. The UK EQUATOR Centre; 2025. Available from: [https:/resources.equator-network.org/reporting-guidelines/care/care-checklist.docx](https://https:/resources.equator-network.org/reporting-guidelines/care/care-checklist.docx)
